# Utilization of peripheral glucose and lactate differences in the diagnosis of feline arterial thromboembolism: a multi-center study

**DOI:** 10.3389/fvets.2024.1505479

**Published:** 2024-12-04

**Authors:** Michael Yee, Julien Guillaumin, Meredith 't Hoen, Jiazhang Cai, Jonathan Mochel, Rebecca Walton

**Affiliations:** ^1^Department of Veterinary Clinical Sciences, Iowa State University, Ames, IA, United States; ^2^Department of Clinical Sciences, Colorado State University, Fort Collins, CO, United States; ^3^Precision One Health Initiative, The University of Georgia, Athens, GA, United States; ^4^VCA West Los Angeles, Los Angeles, CA, United States

**Keywords:** thrombosis, saddle thrombus, cardiomyopathy, hypercoagulability, delta, differential

## Abstract

**Objective:**

To establish lactate and glucose differences between affected and non-affected limbs in cats with feline arterial thromboembolism (FATE). To evaluate the correlation between these values and survival to discharge as well as congestive heart failure (CHF).

**Methods:**

Blood glucose and lactate concentrations were prospectively obtained on admission from client-owned FATE cats and client-owned cats presented for other conditions. The glucose and lactate concentrations of the front limbs (or non-affected) and the hind limbs (or affected) were measured. Lactate (Δlactate) and glucose (Δglucose) differences between the two limbs were calculated.

**Results:**

The FATE group and control groups included 18 and 41 cats, respectively. The median age of the cats within the FATE group and control groups was 9.5 years and 5 years, respectively. The median Δlactate was significantly higher in the FATE group than in the control group (7.2 mmol/L versus 0.1 mmol/L, respectively; *p* ≤ 0.01). The median Δglucose was significantly higher in the FATE group than in the control group (155 mg/dL versus 3 mg/dL, respectively; *p* ≤ 0.01). A diagnosis of FATE was made using a ∆lactate cutoff of 2.2 mmol/L (100% sensitivity and 95% specificity) or a ∆glucose cutoff of 41 mg/dL (100% sensitivity and specificity). There was no correlation between Δlactate and Δglucose and survival to discharge. A higher ∆glucose was significantly associated with concurrent CHF (*p =* 0.01).

**Conclusion:**

Both Δlactate and Δglucose were accurate diagnostic tools for cats with FATE. Neither were correlated with survival to discharge. Higher Δglucose values were significantly associated with the presence of CHF.

## 1 Introduction

Feline arterial thromboembolism (FATE) is a condition involving acute thrombus embolization, most commonly at the aortic trifurcation (1). The development of FATE has primarily been associated with cardiac disease, although other risk factors including systemic diseases such as neoplasia and hyperthyroidism have also been reported (1–4). Regardless of the etiology, FATE results in tissue ischemia, inadequate oxygen supply, and anaerobic glycolysis, ultimately leading to systemic consequences. Clinical signs include unilateral or bilateral paraparesis or paraplegia, pain, vocalization, cyanosis of footpads, absent or diminished arterial pulses, and poikilothermy (1, 5–7). Feline arterial thromboembolism carries a fair to guarded prognosis with approximately 40–50% survival rates being reported when euthanasia at admission is excluded (1, 3, 8).

Prompt diagnosis and treatment are crucial to limit ischemic necrosis and reduce the risk of ischemia reperfusion injury and significantly reduce the risk of complications and amputation of affected limbs (5, 9). However, there is limited literature evaluating the diagnosis of FATE, and a presumptive diagnosis is often made based on the presence of clinical signs. A means of ruling in FATE would be helpful in patients where additional differentials for paralysis, such as orthopedic or neurologic injuries, are being considered (10, 11). Definitive diagnosis can be made using advanced imaging such as angiography or direct thrombus visualization with ultrasound, but these modalities may not be widely available or obtained in a clinically relevant timeframe. Infrared thermography has also been used for diagnosis of FATE; however, it is not readily available in most practices (11). As efficient diagnosis is essential for timely treatment of FATE, an objective, rapid, affordable, bedside diagnostic test would be beneficial. Due to the compromised vascular supply and anaerobic glycolysis that occurs with FATE, lactate production should increase and glucose concentrations should decrease in the affected limbs compared to unaffected limbs (1, 12). Prior studies have investigated the comparative glucose differences in cats and dogs with arterial thromboembolism, but there is limited research evaluating lactate values in cats with FATE (10, 13).

The primary objective of this study was to establish cutoff values to diagnose FATE using differences in lactate (Δlactate) and glucose (Δglucose) blood concentrations between affected and non-affected limbs (objective 1). It was hypothesized that FATE cats would have a Δlactate and Δglucose significantly higher compared to a control population. The secondary objectives of this study were to evaluate the association between Δlactate and Δglucose and survival to discharge (objective 2) and the presence of concurrent congestive heart failure (CHF) (objective 3). It was hypothesized that a large Δlactate and Δglucose would be associated with a lower survival to discharge and higher incidence of CHF.

## 2 Materials and methods

### 2.1 Case selection

Cats diagnosed with FATE were prospectively enrolled from four different institutions between January 2015 and April 2019 as part of a prospective multicenter study which has been published elsewhere (8). Cats were included in that study if they presented within 6 h of onset of clinical signs consistent with FATE and met additional diagnostic criteria supportive of FATE. These cats were included in the present study if glucose blood concentration, lactate blood concentration or both were measured from affected and non-affected limb(s) and Doppler flow was absent in the affected limb. Cats were excluded if FATE was diagnosed only by compatible clinical signs or if no glucose and lactate concentrations were measured. Blood samples from both affected and non-affected limbs were collected via direct venipuncture using a 22-gauge needle and syringe, and analysis was performed within 1 min of sample acquisition. Lactate blood concentrations were measured using point-of-care, handheld lactate meters (Lactate Plus Meter, Nova Biomedical, Waltham, MA). Glucose blood concentrations were measured using point-of-care handheld glucometers (AlphaTRAK Blood Glucose Monitoring System, Zoetis, Parsippany, NJ). Variables collected for all cases included cat signalment, presenting rectal temperature, heart rate, temperature, glucose and lactate levels in affected and non-affected limb(s), Doppler flow, evidence of CHF, and survival to discharge. Cats were determined to have evidence of CHF based on thoracic radiography with interpretation by a board-certified radiologist.

For comparison, 41 cats presenting to the emergency service of two university teaching hospitals were prospectively enrolled as controls. Cats presenting to the emergency service for any condition other FATE were eligible for inclusion. The study was approved by the Institutional Animal Care and Use Committee (IACUC # 3998 for Colorado State University and #22-222 for Iowa State University). Once client consent was obtained, blood samples were collected from the front and hind limbs via direct venipuncture using a 22-gauge needle and syringe. Lactate and glucose analysis was performed within 1 min of sample acquisition according to manufacturer’s recommendations using point-of-care handheld lactate meters (Lactate Plus Meter, Nova Biomedical, Waltham, MA) and glucometers (AlphaTRAK Blood Glucose Monitoring System, Zoetis, Parsippany, NJ), respectively.

### 2.2 Statistical analysis

The primary variable in the statistical analysis was the differences in lactate (Δlactate) and glucose (Δglucose) blood concentrations. The difference in lactate and glucose was defined as affected limb minus non-affected limb in FATE cats and back limb minus front limb in the control group. The analysis included three main objectives: (1) establishing a cutoff for Δlactate and Δglucose to differentiate cats with FATE from cats without FATE (control group); (2) evaluating the association between Δlactate and Δglucose in outcome in cats with FATE; and (3) evaluating the association between Δlactate and Δglucose and CHF in cats with FATE.

For *Objective 1*, the difference between the populations of diseased and healthy cats was evaluated. The normality of the distribution was assessed using the Shapiro–Wilk normality test. Subsequently, the exact two-sample *t*-test was used to compare the two populations. Additionally, we constructed a logistic regression model to determine ATE status based on Δlactate and Δglucose and identified the optimal cutoff for the logistic regression output using ROC (receiver operating characteristic) analysis. Finally, we determined the cutoff for Δlactate and Δglucose by solving the inverse function of the logistic regression. For *Objective 2*, we simplified the survival to discharge, defined as being alive and discharged, or nonsurvival, defined as all other outcomes. The Shapiro–Wilk normality test was performed on the survival and non-survival populations, followed by the Kruskal-Wallis rank sum test and the exact two-sample *t*-test to compare differences between populations. *Objective* 3 followed a similar analysis process as Objective 2. *p*-values < 0.05 were considered statistically significant. The statistical analysis was conducted using R software version 4.3.1, and graphical representations of the data were produced using the ggplot2 package in R version 4.3.1 (14).

## 3 Results

Thirty-two cats were prospectively enrolled and diagnosed with FATE; however, 14 were excluded based on lack of diagnosis made with Doppler flow. Therefore, 18 cats were included in the analysis. Most cats were Domestic Short Hair (12); other breeds included European Shorthair (2), Maine Coon (1), Sphinx (1), Domestic Medium Hair (1), and Bengal (1). The median age was 9.5 years (range 1.5–15 years), and the median weight was 5.1 kilograms (range 2.3–8.14 kilograms). Fifteen cats were castrated males, and three cats were spayed females. A total of 16/18 (89%) cats were paraparetic/paraplegic on presentation, 1/18 (5.5%) was monoparetic and 1/18 had three limbs affected. The median rectal temperature on presentation was 96.9° F (range 90–102.2°F; reference interval 100.5–102.5°F). The median heart rate on presentation was 200 beats per minute (bpm) (range 140–280 bpm; reference interval 160–220 bpm), and the median respiratory rate was 60 breaths per minute (range 36–120 breaths per minute; reference interval < 40 breaths per minute). A total of 13 cats (72%) were diagnosed with CHF based on thoracic radiographs. Five cats (28%) survived to discharge, and 13 cats (72%) did not survive to discharge. Eight cats were euthanized, and five cats died naturally.

In FATE cats, the median lactate of an unaffected limb was 3.5 mmol/L (range 2.2–5.7 mmol/L; reference interval < 2.5 mmol/L). The median lactate value of an affected limb was 11.5 mmol/L (range 5.2–17 mmol/L; reference interval < 2.5 mmol/L). The median ∆lactate of the affected and unaffected limbs was 7.2 mmol/L (range 0.3–14.8 mmol/L). The median blood glucose of the unaffected limb was 250 mg/dL (range 112–430 mg/dL). The median glucose of the affected limb was 103 mg/dL (range 26–185 mg/dL). The median ∆glucose of the affected and unaffected limbs was 155 mg/dL (range 47–322 mg/dL) (Table 1).

**Table 1:** The calculated lactate and glucose differences between affected and non-affected limbs in cats with feline arterial thromboembolism (FATE) compared to control cats without arterial thromboembolism.

	FATE group (*n* = 18)	Control group (*n* = 41)
∆lactate (mmol/L)	7.2	0.1
∆glucose (mg/dL)	155.0	3.0

The control group was comprised of 41 cats that were prospectively enrolled. Most cats were Domestic Short Hair (35); other breeds included Domestic Medium Hair (4) and Domestic Long Hair (2). Twenty-eight of the cats were neutered males and 13 of the cats were spayed females. The median age was 5 years (range 1–11 years old). Cats in the control group presented to the emergency service for a variety of complaints including urinary obstruction (9), gastrointestinal signs (8), acute kidney injury (4), orthopedic injuries including fractures or lameness (3), heart disease (3), toxicoses (2), vomiting (2), seizures (2), anal gland abscess (2), septic peritonitis (2), and one of each for epistaxis, nasal discharge, trauma and respiratory distress. No control cat was diagnosed with CHF or FATE. In the control group, the median lactate of the thoracic limbs was 1.7 mmol/L (range 0.4–6.1 mmol/L; reference interval < 2.5 mmol/L) and the median lactate of the pelvic limbs was 1.5 mmol/L (range 0.7–4.4 mmol/L). The median Δlactate was 0.1 mmol/L (range −1.8 to 1.6 mmol/L). The median glucose of the thoracic limbs was 112 mg/dL (range 60–243 mg/dL), and the median glucose of the pelvic limbs was 116 mg/dL (58–251 mg/dL). The median ∆glucose was 3 mg/dL (range −100 to 41 mg/dL) (Table 1).

There was a significant difference between the ∆lactate and ∆glucose of the FATE and control groups (*p* ≤ 0.01). The use of ROC curve analysis showed the optimal ∆lactate cutoff of 2.2 mmol/L resulted in a diagnosis of FATE with 100% sensitivity and 95% specificity, with a positive predictive value (PPV) of 86% and negative predictive value (NPV) of 100% (Table 2). The ∆lactate was not significantly different between cats that were diagnosed with CHF, compared to those without, and ∆lactate was not significantly different between cats that survived to discharge and those that did not (*p =* 0.87 and 0.12, respectively).

**Table 2:** Cutoff values for ∆lactate and ∆glucose between affected and non-affected limbs in cats with feline arterial thromboembolism and their associated sensitivity, specificity, positive predictive value (PPV) and negative predictive value (NPV).

	∆lactate cutoff of 2.2 mmol/L	∆glucose cutoff of 41 mg/dL
Sensitivity	100%	100%
Specificity	95%	100%
PPV	86%	100%
NPV	100%	100%

The use of ROC curve analysis showed the optimal ∆glucose cutoff of 41 mg/dL resulted in a diagnosis of FATE with 100% sensitivity and specificity with a PPV and NPV of 100% (Table 2). ∆glucose was significantly associated with a diagnosis of CHF (*p =* 0.01), and cats with a higher ∆glucose were more likely to be diagnosed with CHF. The ∆glucose was not significantly associated with outcome (*p =* 0.4).

## 4 Discussion

This study aimed to determine a Δlactate and Δglucose that would aid in rapid identification of FATE. A previous study, published as an abstract, noted higher local venous lactate concentrations and lower glucose concentrations of limbs affected by FATE, compared to central venous values; however, no cutoff was established (13). Our results demonstrated a Δlactate of 2.2 mmol/L or a Δglucose of 41 mg/dL has excellent diagnostic performance in the diagnosis of FATE compared to cats with non-FATE related emergency presentations. While Δlactate was not associated with a diagnosis of CHF or outcome, Δglucose was significantly associated with a diagnosis of CHF, but not outcome.

Our study is the first to report an optimal cutoff for ∆lactate for the diagnosis of FATE. Previously reported lactate concentrations in dogs and cats with ATE were significantly (*p* = 0.001) higher in venous samples of the affected limb (10.7 ± 2.7 mmol/L) compared to central venous samples (2.1 + 0.8 mmol/L). The reported values in affected and non-affected limbs were similar to those in our study, which reported a median lactate of 11.5 mmol/L and 3.5 mmol/L in the affected and non-affected limbs, respectively. However, both dogs and cats with ATE were included in that study and an optimal lactate cutoff was not provided (13).

An optimal Δglucose of 41 mg/dL for diagnosis of FATE was identified in this study. This is close, but not identical, to the 30 mg/dL Δglucose cutoff identified in a previous study (10). Our study had an improved sensitivity and specificity (100%) compared to a sensitivity of 100% and a specificity of 90% in that same study (10). Those differences may be explained by the fact that, although our study had a similar number of FATE cats, FATE cats were from multiple institutions, our control group was more robust, and our study used stricter criteria for the diagnosis of FATE, requiring lack of Doppler flow compared to clinical signs only (10). It has been demonstrated that venous glucose concentrations in the affected limb are significantly lower than the central glucose concentrations in thromboembolism, but the results include both dogs and cats and do not provide an optimal cut-off value (13).

A median glucose of 103 mg/dL and 250 mg/dL in the affected and non-affected limbs, respectively, was demonstrated in this study. These values are higher as compared to prior studies, which reported a median glucose of 45–50 mg/dL in the affected limb and 181-182 mg/dL in the non-affected limb (10, 13). Compared with prior studies, our study only enrolled cats showing clinical signs of FATE within 6 h of presentation as opposed to within 24 h. The difference in time to presentation may explain the differences in blood glucose in cats with FATE versus non-FATE cats, as glucose of the affected limb is expected to decrease with prolonged ischemia. Additionally, the decreased time to presentation may have allowed these patients to experience a more profound stress response resulting in a higher peripheral glucose.

The clinical signs of FATE are due to acute ischemic injury, localized to the aortic trifurcation in up to 71% of cases (1). Prompt diagnosis of FATE is necessary for identification of underlying conditions and proper treatment, including analgesia and antithrombic therapy (8). Diagnostic criteria for FATE include physical exam findings such as pulselessness, pain, pallor, paresis and poikilothermia; however, confirming FATE as the cause of appendicular signs may be challenging in some cats (1). Pulselessness may be suspected in cats with difficult palpation, including obese or uncooperative cats, and poor or absent pulses may also be due to systemic hypotension (1). Other diagnostic techniques include Doppler flow and thermal imaging, however, each has potential limitations. Doppler blood pressure may be difficult to obtain and infrared thermography is not routinely available and may be cost prohibitive.

Concurrent CHF has been reported in 40–66% of cats diagnosed with FATE (1, 3, 7). Cats presenting with FATE with and without CHF can be difficult to differentiate based on physical exam findings and respiratory rate on presentation, as considerable overlap in respiratory rate has been noted in cats with and without CHF (1). Treatment of CHF is imperative, and diagnosis often requires additional testing such as thoracic radiographs, which may not be feasible during the initial presentation. This study aimed to evaluate the association between Δlactate and Δglucose and a diagnosis of CHF in cats with FATE, as rapid diagnosis may aid initial management strategies. In humans with CHF, hyperglycemia (defined as 144–180 mg/dL) is common due to impaired insulin mediated glucose uptake and cytokine mediated suppression of insulin release and has been shown to be a negative prognostic indicator (15, 16). Similar studies in dogs demonstrated an association between hyperglycemia and a worse outcome with CHF (17); however, no studies in cats have evaluated this association. Our study demonstrated an increase in Δglucose was associated with a diagnosis of CHF and large Δglucose values should increase clinical suspicion for concurrent CHF.

In contrast to Δglucose, an increase in Δlactate values were not associated with a diagnosis of CHF. Hyperlactatemia, defined as lactate >2.5 mmol/L, has been demonstrated in dogs with CHF and is presumed to be secondary to increased tissue hypoperfusion. The degree of hyperlactatemia has also been shown to correlate directly with severity of disease (18). Cats with FATE and CHF may be hyperlactatemic secondary to tissue hypoperfusion as well as regional hyperlactatemia in the ischemic limb. Therefore, Δlactate may not be significantly increased in cats with both FATE and CHF.

The survival to discharge in this study (28%) is similar to previously reported survival rates (1). The prognosis of FATE is fair to guarded, often because it occurs secondary to a significant underlying disease, while also resulting in severe hemodynamic compromise (1). Significant differences have been noted for rectal temperature and heart rate between survivors and nonsurvivors (6). Values for Δlactate and Δglucose have not been previously evaluated in survivors versus nonsurvivors. The results of this study showed no association between Δlactate and Δglucose levels and survival to discharge. This may be due to the high rate of euthanasia, patient selection, or the study sample size.

There are several limitations to this study. Our study is limited by a small sample size, but the use of a prospective data set and a robust 1:2 patient to control group compensates for this limitation. Another limitation is that the FATE group only included cats with confirmed diagnosis based on lack of Doppler flow at presentation. While absence of Doppler flow was used in diagnosis of cats with FATE, the gold standard diagnosis for FATE is with contrast media angiography or direct visualization of the thrombus. Inclusion of cats that were diagnosed with FATE based on absence of Doppler flow may have impacted patient selection, as sicker cats may have been diagnosed on clinical signs alone without additional testing and would not have been included in this study. Regarding objectives 2 and 3, our study may have been underpowered to detect a difference in survival and CHF. Regarding objective 2, eight of the 13 cats that did not survive to discharge were euthanized. Some of the euthanized patients may have ultimately survived impacting overall outcomes. Another limitation is that our study utilized a set of specific glucometers and lactate meters. Though the differences in analyzers should be minimal there may be differences in Δlactate and Δglucose cutoffs depending on the analyzers utilized.

In conclusion, Δlactate and Δglucose are readily available, affordable, easily performed bedside diagnostics with a high sensitivity and specificity for a diagnosis of FATE. Additionally, Δglucose is associated with a diagnosis of CHF and may be utilized in prioritizing initial patient intervention and diagnostics.

## Data Availability

The raw data supporting the conclusions of this article will be made available by the authors, without undue reservation.
